# Supplementary material for: Pathogen transmission from vaccinated hosts can cause dose-dependent reduction in virulence
Source: PLoS Biol. 2020 Mar 5;18(3):e3000619. doi: 10.1371/journal.pbio.3000619 (PMC7058279; doi:10.1371/journal.pbio.3000619)
Supplement: S1 Text — (DOCX) [file pbio.3000619.s001.docx]

**Pilot study design summary.** The design and sampling protocol of the MD transmission experiment presented in the main manuscript builds on results from previous small-scale pilot experiments to establish (i) the appropriate contact duration between shedder and contact birds required for successful virus transmission, (ii) first estimates for the onset and duration of the infectious period of shedder birds and the impact of vaccination on it, and (iii) the appropriate time for collecting feather samples for detecting virus in contact birds infected by shedder birds. All pilot experiments were carried out in the same facilities as the main experiment (Avian Disease and Oncology Laboratory, ADOL, East Lansing, MI USA). Unless otherwise stated, the same experimental protocol was adopted for all experiments.
