# Supplementary material for: Pathogen transmission from vaccinated hosts can cause dose-dependent reduction in virulence
Source: PLoS Biol. 2020 Mar 5;18(3):e3000619. doi: 10.1371/journal.pbio.3000619 (PMC7058279; doi:10.1371/journal.pbio.3000619)
Supplement: S2 Text — (DOCX) [file pbio.3000619.s002.docx]

**Determination of the duration of contact between shedder and contact birds required for successful virus transmission.** In this pilot experiment, contact birds came from the same chicken line as in the main experiment (maternal antibody negative 15I_5_ x 7_1_ F_1_), while shedders belonged to the inbred white leghorn chicken Lines 6_3_ (resistant to MD) and 7_2_ (susceptible to MD), all developed at the ADOL [1]. For each replicate, 28 newly hatched contact birds were placed with three-week old shedder birds from either line 6 or 7. Contacts were randomly allocated into four groups (hence 4x28=112 contact birds total). Subsequently, on each of hours 4, 8, 12, 24, 48, 96 and 168, four contact birds from each replicate were removed and placed together in an isolator (different isolator for each replicate, i.e., 4 birds per isolator). Isolated contacts were then monitored for 8 weeks and necropsied to determine MD status. Hence there were two independent groups of four contacts per shedder line per time period.

The proportion of contact individuals showing visible disease symptoms upon necropsy at 8 weeks post-contact was universally high in this experiment (S1 Fig). Hence, it was concluded that 48 hours of contact is sufficient to establish MDV transmission from shedders with both high and low MD genetic resistance.

**S1 Fig. Effects of exposure duration on contact bird Marek’s disease.** For each tested contact duration, the proportion of line 15I_5_ x 7_1_ F_1_ contact birds positive for Marek’s disease symptoms at necropsy, 8 weeks post-contact with inoculated unvaccinated “MD-resistant” line 6 (blue line) or “MD-susceptible” line 7 (red line) shedder birds.

**Reference**

1. Vallejo RL, Bacon LD, Liu HC, Witter RL, Groenen MA, Hillel J, Cheng HH. Genetic mapping of quantitative trait loci affecting susceptibility to Marek's disease virus induced tumors in F2 intercross chickens. Genetics. 1998 Jan 1;148(1):349-60.
