# Supplementary material for: Pathogen transmission from vaccinated hosts can cause dose-dependent reduction in virulence
Source: PLoS Biol. 2020 Mar 5;18(3):e3000619. doi: 10.1371/journal.pbio.3000619 (PMC7058279; doi:10.1371/journal.pbio.3000619)
Supplement: S3 Text — MDV, Marek disease virus. (DOCX) [file pbio.3000619.s003.docx]

**Establishing the onset and duration of the infectious period of vaccinated and sham-vaccinated MDV-infected shedder birds.** We carried out two replicates of experiments to examine whether vaccination affects the onset and duration of MDV transmission of infected shedder birds, and whether this is reflected by differences in shedder feather viral load over time. Groups of 3 shedder individuals of the same vaccination status were placed in contact with new independent groups of 10 contact birds every 2 days from 10-20 DPI, and recording of contact bird infection (qPCR at 14 DPC) and disease (necropsy at 8 weeks post-contact) status was carried out. The same inbred chicken line as the main experiments, 15I_5_ x 7_1_ F_1_, was used for both shedders and contacts, and protocols otherwise matched those of the main experiment. Shedder FVL was recorded at the beginning of each contact period, as well as at 7 DPI and weekly from days (DPI) 21-56 (S2 Fig).

Inspection of FVL profiles fitted to the repeated FVL measures of vaccinated and non-vaccinated shedders (S2 Fig) showed that vaccinated shedders had consistently lower FVL than sham-vaccinated shedders and that both types of birds experienced an initial sharp rise in FVL. However, in sham-vaccinated shedders this initial sharp rise started to plateau at around 11-12 DPI, whereas FVL reached a peak at around 20 DPI in vaccinated shedders (S2 Fig). The profiles suggest that any FVL-mediated effects of shedder vaccination on contacts should differ more at the earlier DPI. Given these results, DPI 13 and 20 were chosen as the most informative time points to investigate vaccination effects on MDV transmission and subsequent disease progression in contact birds in the main experiment.

**S2 Fig. Feather viral load over time for shedder birds.** Vaccinated (blue) and sham-vaccinated (red) shedders, with maximum likelihood broken stick regression lines indicating lower viral load and a later breakpoint in viral load over time for vaccinated shedders. Open circles = replicate 1, crosses = replicate 2. The shaded area encompasses the set of shedder DPI during which contact occurred between shedders and contact birds.

Almost all contacts became infected regardless of shedder DPI, indicating that shedder birds started to become infectious prior to 10 DPI and remained infectious until at least 20 DPI (S3 Fig). A much lower proportion of contact birds developed disease symptoms or died when exposed to vaccinated shedders. Results were also suggestive that shedder DPI at exposure may have some influence on disease progression in contact birds: more contact birds developed disease symptoms when exposed to vaccinated shedders from 14 shedder DPI onwards, and more contact birds died after contact with sham-vaccinated shedders at later shedder DPI.

**S3 Fig. Impact of shedder vaccination status and days post-infection on contact bird infection, disease symptoms and mortality.** Contacts positive for virus in qPCR from samples taken at 14 DPC were classified as infected. “Diseased” individuals showed visible symptoms (peripheral nerve enlargement and/or tumours) at necropsy, 8 weeks post contact or upon death. “Dead” individuals died due to MD prior to the end of the 8-week experimental period. HVT = contacts exposed to vaccinated shedders; PBS = contacts exposed to sham-vaccinated shedders. The two replicates were pooled for this figure.
