# Supplementary material for: Pathogen transmission from vaccinated hosts can cause dose-dependent reduction in virulence
Source: PLoS Biol. 2020 Mar 5;18(3):e3000619. doi: 10.1371/journal.pbio.3000619 (PMC7058279; doi:10.1371/journal.pbio.3000619)
Supplement: S4 Text — FVL, feather viral load. (DOCX) [file pbio.3000619.s004.docx]

**Determination of appropriate sampling time post-contact for measuring FVL in contact birds.** In the main study, FVL was used as a means to establish successful MDV transmission from shedder to contact birds and to gain insights into the potential mechanisms underlying vaccine effects. To avoid confounding between shedder-contact bird and contact-contact bird transmission, FVL samples of contact birds needed to be taken prior to the onset of contact-contact bird transmission.

Two replicates of experiments carried out with unvaccinated shedders of 4 different ADOL inbred chicken lines (MD-resistant Line 6 and Line 15.N-21, and MD-susceptible Line 7 and Line 15.P-19) revealed that 15I_5_ x 7_1_ F_1_ strain contact bird FVL was low and often below the level of detection at 7 DPC, but significantly higher and above the level of detection at 14 DPC (S4 Fig). Hence, for the main experiment FVL in contact birds was measured at 14 DPC, as this provided a reliable indicator of successful transmission between shedder and contact birds, and because 14 DPC lies within the expected period prior to the onset of contact-contact bird transmission and presence of virus in feathers (given an assumed latency period of 7 days [1]).

**S4 Fig. Effect of number of days post-contact on contact bird feather viral load.** Histogram of contact bird FVL from qPCR at 7 (red bars) and 14 (blue bars) days post-contact with unvaccinated infectious shedders (2 replicates and all 4 shedder chicken lines combined). A value of -5 indicates negative for MDV, i.e. values were below the level of detection by standard qPCR.

**Reference**

1. Baigent SJ, Smith LP, Currie RJ, Nair VK. Replication kinetics of Marek's disease vaccine virus in feathers and lymphoid tissues using PCR and virus isolation. Journal of General Virology. 2005 Nov 1;86(11):2989-98.
