# Supplementary figures and images for: Pathogen transmission from vaccinated hosts can cause dose-dependent reduction in virulence
Source: PLoS Biol. 2020 Mar 5;18(3):e3000619. doi: 10.1371/journal.pbio.3000619 (PMC7058279; doi:10.1371/journal.pbio.3000619)

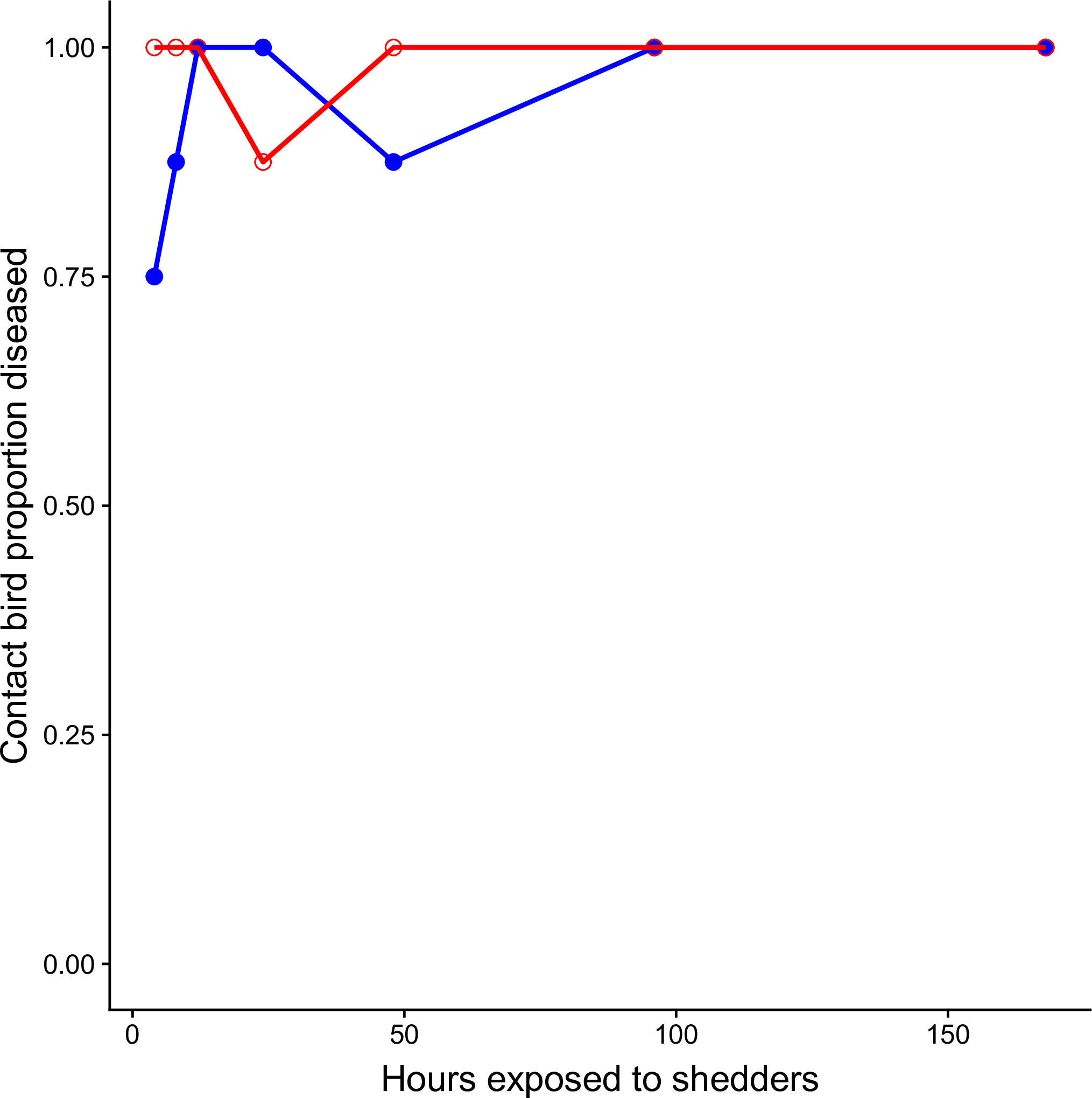

Supplement: S1 Fig — For each tested contact duration, the proportion of line 15I5 × 71 F1 contact birds positive for MD symptoms at necropsy, 8 weeks post-contact with inoculated unvaccinated ‘MD-resistant’ line 6 (blue line) or ‘MD-susceptible’ line 7 (red line) shedder birds. MD, Marek disease. (TIF) [file pbio.3000619.s005.tif]

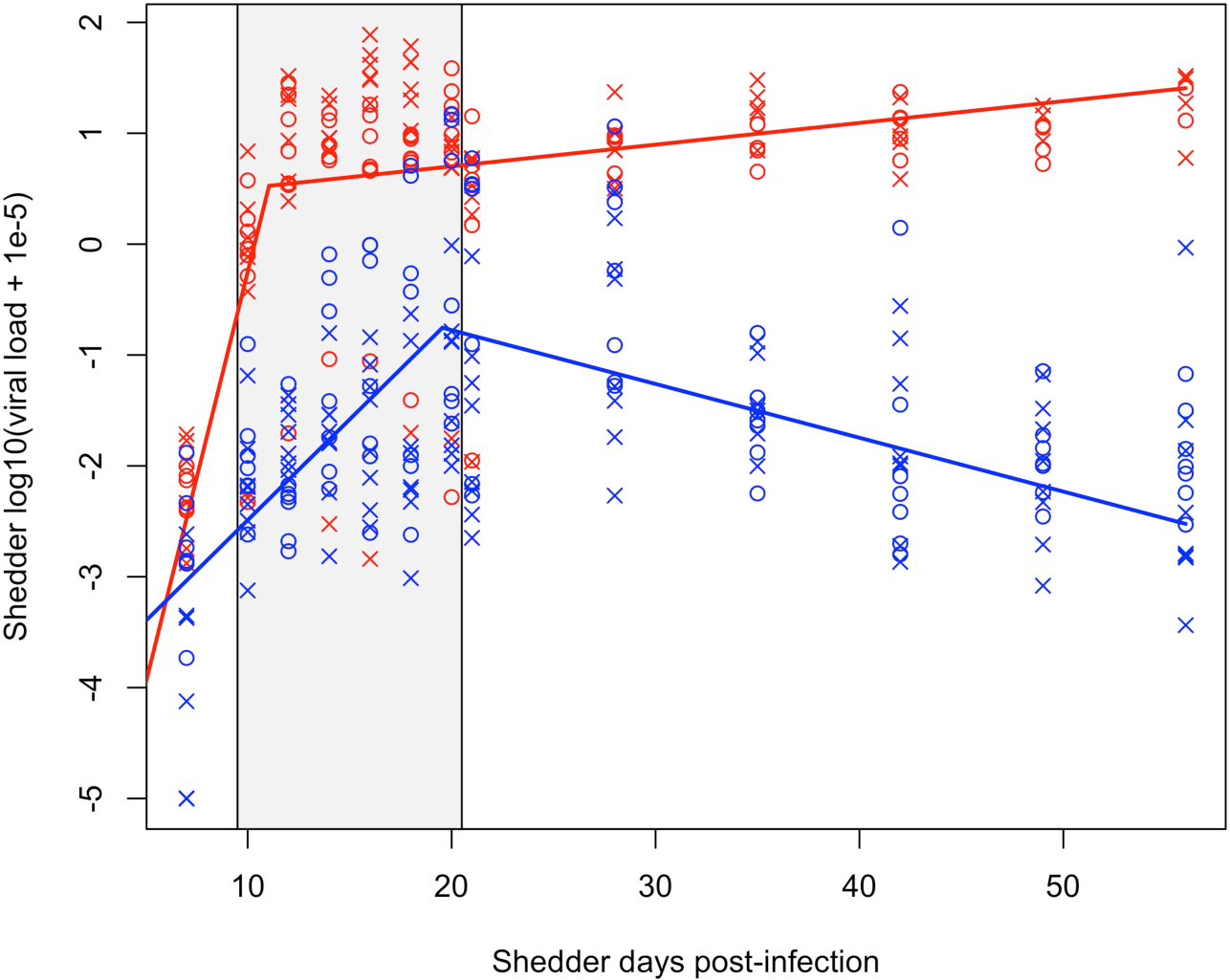

Supplement: S2 Fig — Vaccinated (blue) and sham-vaccinated (red) shedders, with maximum likelihood broken stick regression lines indicating lower viral load and a later breakpoint in viral load over time for vaccinated shedders. Open circles = replicate 1, crosses = replicate 2. The shaded area encompasses the set of shedder DPI during which contact occurred between shedders and contact birds. DPI, days post-infection; FVL, feather viral load. (TIF) [file pbio.3000619.s006.tif]

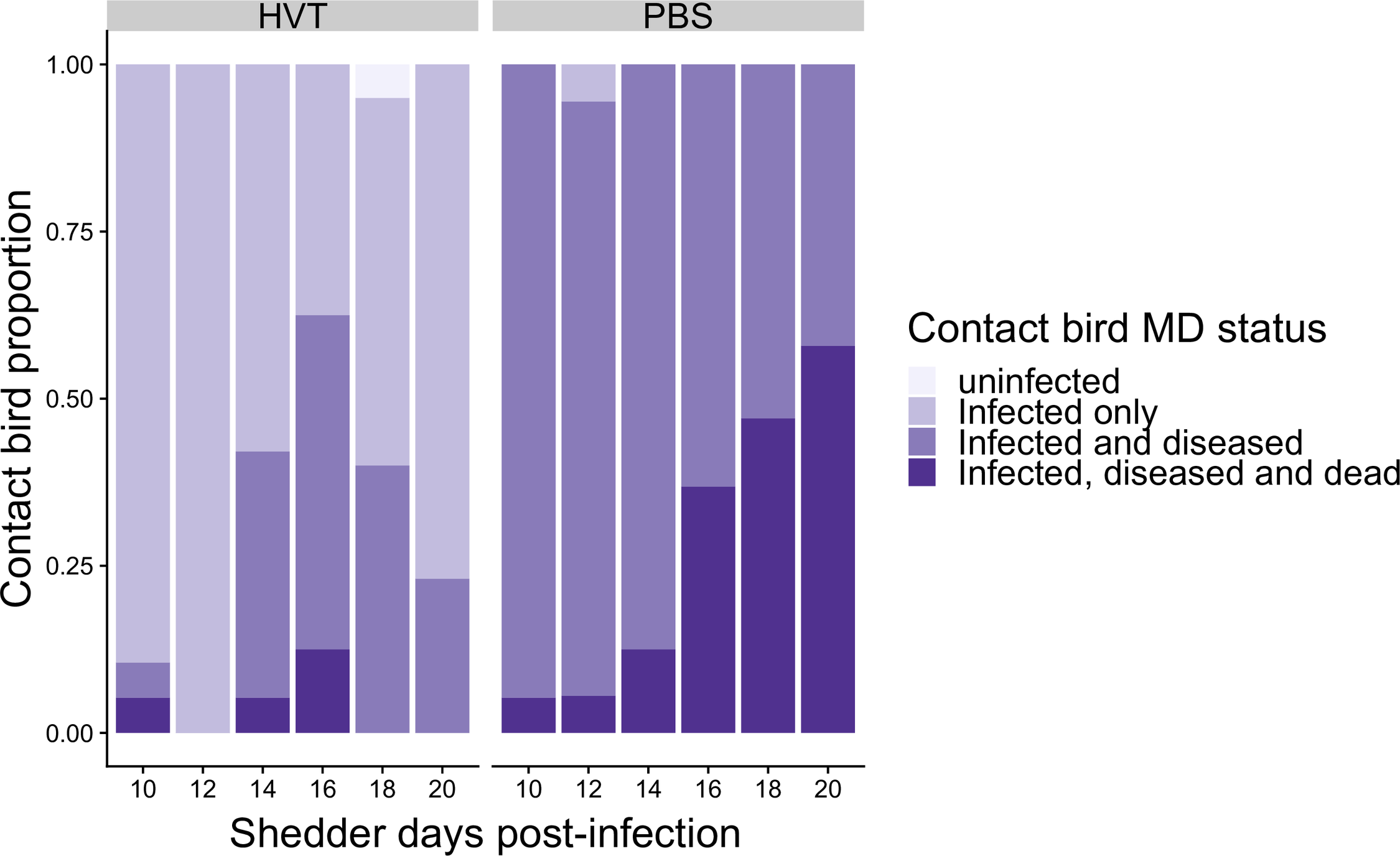

Supplement: S3 Fig — Contacts positive for virus in qPCR from samples taken at 14 DPC were classified as infected. ‘Diseased’ individuals showed visible symptoms (peripheral nerve enlargement and/or tumours) at necropsy, 8 weeks post-contact or upon death. ‘Dead’ individuals died because of MD prior to the end of the 8-week experimental period. HVT = contacts exposed to vaccinated shedders; PBS = contacts exposed to sham-vaccinated shedders. The 2 replicates were pooled for this figure. DPC, days post-contact; DPI, days post-infection; HVT, herpesvirus of turkeys; MD, Marek disease; qPCR, quantitative polymerase chain reaction. (TIF) [file pbio.3000619.s007.tif]

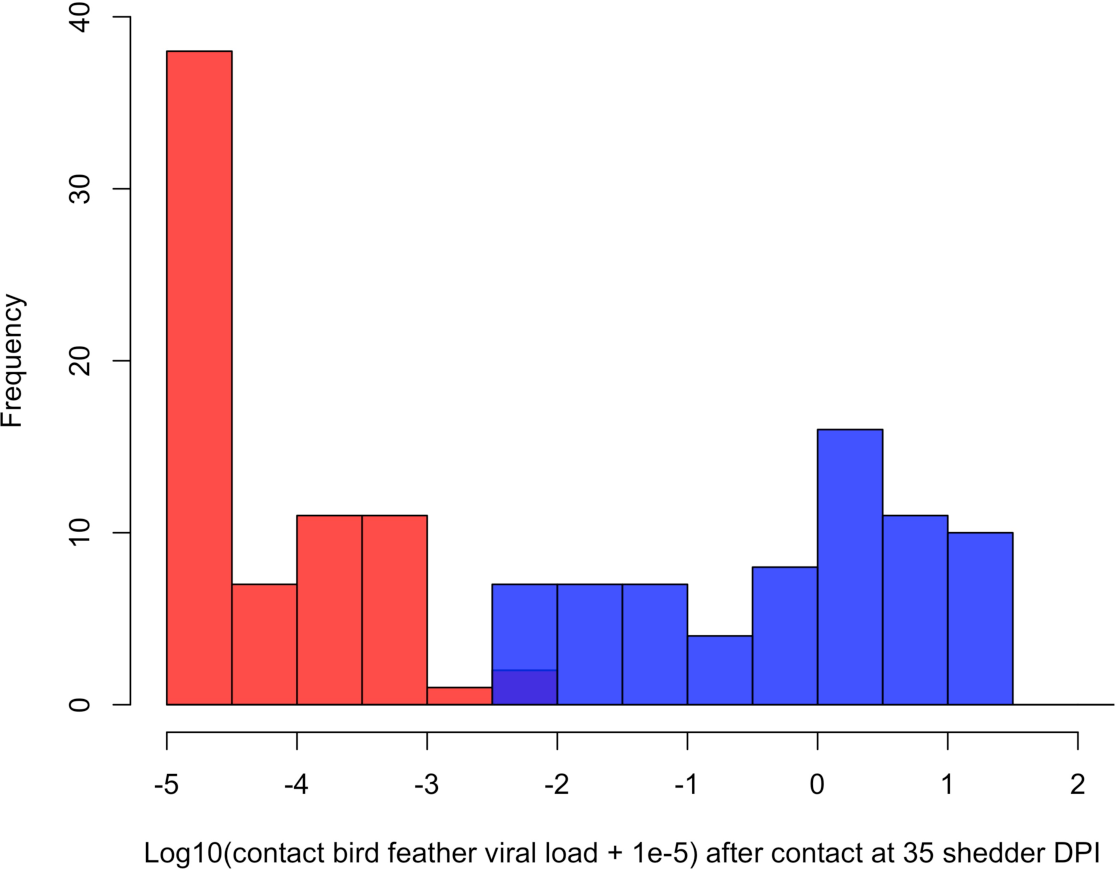

Supplement: S4 Fig — Histogram of contact bird FVL from qPCR at 7 (red bars) and 14 (blue bars) days post-contact with unvaccinated infectious shedders (2 replicates and all 4 shedder chicken lines combined). A value of −5 indicates negative for MDV; i.e., values were below the level of detection by standard qPCR. FVL, feather viral load; MDV, Marek disease virus; qPCR, quantitative polymerase chain reaction. (TIF) [file pbio.3000619.s008.tif]
