# Supplementary material for: Pathogen transmission from vaccinated hosts can cause dose-dependent reduction in virulence
Source: PLoS Biol. 2020 Mar 5;18(3):e3000619. doi: 10.1371/journal.pbio.3000619 (PMC7058279; doi:10.1371/journal.pbio.3000619)
Supplement: Attachment — Submitted filename: PBIOLOGY-D-19-02539R1_ResponseToReviewers_05Dec2019.docx [file pbio.3000619.s010.docx]

Response to reviewers for manuscript PBIOLOGY-D-19-02539R1 entitled “**Transmission from vaccinated hosts can cause dose-dependent reduction in pathogen virulence**”.

Dear Dr Richardson,

Please find below our responses to the Academic Editor and to reviewer comments. Responses begin with the word “Response” in bold. Text from the original PLoS Biology decision email is within quotation marks.

We thank the editors and reviewers for their insightful and constructive comments. We have taken these on board and carefully addressed each one, carrying out further analyses and making substantial revisions to the manuscript. We believe these revisions have significantly improved our paper.

---------------

“As you will read, the reviewers appreciated many aspects of your work. However, they do raise some concerns that will need to be addressed in a revision. Of particular importance, two of the reviewers believe that since the HVT vaccine is also shed into the environment from vaccinated animals, this must be accounted for in your studies. Additionally, the Academic Editor recommends that you extend your discussion of how this work relates to the previous studies on virulence evolution in Marek’s disease.”

**Response**: We have now extended the discussion of virulence evolution as requested by the Academic Editor, incorporating more specific expectations of the impacts of our findings on optimum virulence; lines 425-451.

Please see our responses to reviewers below regarding potential transmission of HVT vaccine virus.

Over and above our responses the Academic Editor and reviewers (below), we have made a few small grammatical changes throughout the manuscript, and fixed a small number of typographical errors. These can be seen in the Track Changes version of the manuscript.

Reviewer #1:

“In this paper, the authors report results of experiments testing for downstream impacts of vaccination by evaluating disease severity in chickens infected by chickens that received the leaky Marek’s vaccine relative to those infected by chickens that received a sham control. Chickens that were infected by vaccinated birds experienced reduced symptoms, and this effect was shown to result from reduced feather virus load in vaccinated birds. The authors argue that these results demonstrate leaky vaccines may be more protective than generally assumed. I enjoyed reading this clear and well-written paper and believe it will stimulate further research into the downstream impacts of vaccination.

Specific comments:

1. The title should be changed to: “Pathogen transmission from vaccinated hosts…” as the original title strongly suggests the paper focuses on transmissible vaccines and is misleading. “

**Response**: The title now reads “Pathogen transmission from vaccinated hosts can cause dose-dependent reduction in virulence”.

“2. The figures appear blurry, at least in my copy.”

**Response**: We have improved the resolution of Fig 1. The others should be publication standard.

“3. In figure 2B. After staring at this figure for a while and repeatedly reading the legend, I do not understand what message it is intended to convey. Also, what explains the odd clustering of points into discrete bands? Is each odd band/cluster a replicate?”

**Response**: the legend for Fig 2B has now been re-worded to improve clarity; lines 209-219. In particular, we now describe the causes of the point-clustering (each cluster is a different number of tumours and/or a different combination of tissues containing tumours), and highlight that the directions of the grey arrows indicate whether variables are positively (same direction) or negatively (opposite direction) associated.

---------------

Reviewer #2:

“Both MDV1 and HVT (vaccine strain) are shed from the infected or vaccinated birds, respectively. It is known that the levels of HVT shedding are increased by 38 folds when the HVT vaccinated birds are infected with MDV1 (Islam A, Walkden-Brown SW. J Gen Virol 2007).

It is conceivable that HVT (as well as MDV1) is transmitted from these birds to the unvaccinated contact birds. Transmission of HVT to the unvaccinated contact birds may inhibit disease progression (unwanted vaccination). Therefore, both lower MDV1 dose as well as possibility of HVT transmission to the unvaccinated birds may be involved in the lack of clinical signs of the disease in spite of virus replication.

No attempt has been made to examine the role of HVT transmission to the unvaccinated contact groups, and analyze its effects on the positive consequences in controlling the spread ad symptoms of the disease.”

**Response.** We thank reviewers 2 and 3 for highlighting the possibility of HVT transmission, and we agree that this is an important topic to address. We believe discussion of this topic further adds value to our manuscript for a wide audience. We address the general issue raised by both reviewers here, and separately address the additional studies requested by reviewer 3 below.

There is indeed literature evidence that HVT is shed into the environment (Islam et al 2005, 2007), although these authors indicated their belief that transmission is nevertheless unlikely. Indeed, earlier studies specifically examining transmission (Cho 1976; Cho & Kenzy 1973, 1975), testing a variety of scenarios, showed that HVT vaccination of young birds, at low dose (similar to the 2,000 PFU in our study, which itself is a quarter of the 8,000 PFU used in Islam et al 2005, 2007), led to zero HVT transmission. Therefore, *a priori* we felt it reasonable to assume no HVT transmission. In fact, this was one of the primary reasons we chose HVT over other vaccines.

However, given the strength of the downstream effects revealed in our studies, we acknowledge a possible influence of HVT transmission in contributing to these effects, and we also acknowledge the relevance to our paper of a broader discussion of vaccine transmission. Therefore, as well as now discussing more broadly the possible impacts of live vaccine transmission in the Introduction (lines 114-121) and Discussion (lines 438-455), we carried out (1) additional qPCR to quantify HVT virus in a subsample of contacts of vaccinated shedders, and (2) new nested regression analyses, to test whether the very strong relationships between contact bird disease variables and contact bird feather pathogen load remain significant within each shedder vaccination treatment. Vaccine virus may independently affect pathogen viral load and disease development, and alter the relationship between the two. Hence, the nested analysis was aimed to show whether these relationships were similar and equally strong in the presence and absence of potential HVT transmission. Results and interpretation are described below.

We evaluated a subsample of 89 blood samples (buffy coats) from contact birds originating from 3 replicates with 100% survival, as well as from 3 replicates with the lowest survival (39-67% survival). Only 9% (8/89) of contact birds in our subsample were positive for HVT in blood (see Methods lines 620-635 and lines 688-690, and Results lines 355-368), and there was no correlation based on percent survival of the replicate. Although this shows for the first time that HVT virus can be transmitted from young birds vaccinated with low doses, the fact that ~90% of individuals were negative indicates that this transmission is not sufficient to explain the downstream vaccine effects. Unless undetectable levels of HVT still confer considerable protection, the most parsimonious conclusion from this result alone is that HVT transmission cannot be the primary explanation for our results. We therefore did not explicitly incorporate HVT transmission into our statistical process analysis.

Our nested regression analyses provide more concrete support for a direct causal relationship between pathogen load and disease progression, and for the absence of any major influence of HVT transmission. For all contact bird disease variables that showed significant positive relationships with same-individual feather viral load in existing analyses (i.e. all response variables except vagus nerve enlargement, see Table S1), this significant relationship remained and with similar effect sizes within both contacts of vaccinated and contacts of unvaccinated shedders (see Results lines 399-405 and Table S1). HVT transmission cannot contribute to this relationship within the unvaccinated shedder treatment. We highlight this in the Discussion (lines 451-455).

As we already state in the Results (lines 389-392) and detail in Table S1, our proxy for exposure dose - shedder feather viral load (FVL) - does not always fully explain the shedder vaccination effect on contact bird disease progression, leaving open the possibility of another unmeasured factor contributing to the downstream effects of vaccination. On the other hand, shedder FVL at the start of a 48-hour contact period may simply be an imperfect proxy for exposure dose. We acknowledge the utility of future studies to disentangle these possibilities, while reiterating that they do not undermine our results, in particular given that the within-individual relationship between FVL and disease is unaffected. We have included mention of this specific point in the new Discussion paragraph on possible vaccine virus transmission (lines 445-455).

---------------

Reviewer #3:

“General Comment:

In the submitted manuscript, the authors investigated the effect of leaky vaccines on the transmission of an avian herpesvirus to unvaccinated contact chickens. They claim that the reduced replication of Marek’s disease virus (MDV) in vaccinated animals has a protective effect on the contact chickens. While this information would be very exciting to a broad audience, their experiments do not provide sufficient convincing evidence to support this conclusion (yet). For example, the authors completely disregard the fact that the HVT vaccine is also shed into the environment and could therefore provide some protection to the contact animals. This and other aspects are outlined in the major and minor points below.

Major Points:

1)	The main message of the manuscript is that the use of leaky vaccines reduces the severity and frequency of clinical symptoms of Marek’s disease virus (MDV) in contact chickens. Unfortunately, the author completely disregard the fact that this leaky HVT vaccine is also shed into the environment from vaccinated animals, and also upon challenge with MDV [1, 2]. Therefore, the contact animals likely acquired HVT that in turn would provide some protection against MDV. In poultry industry, animals are vaccinated just hours before they get exposed to MDV in a contaminated environment, confirming that a concurrent vaccination can efficiently provide protection.”

**Response**: We thank the reviewer for emphasizing the need to explore the possibility of HVT transmission, and acknowledge that this was a missing piece in our submitted manuscript. Please see our response to reviewer 2 above for details of how we have dealt with this issue. Our new results clarify that HVT transmission is not the primary determining factor influencing the downstream impacts on contact bird disease progression. We cannot rule out the possibility that the presence of even undetectably small quantities of HVT induced some level of protective response in unvaccinated contact birds, and indeed, the lower viral loads in contacts of vaccinated shedders could still be a consequence of HVT transmission. But we reiterate that the evidence we present strongly supports a direct causal relationship between pathogen load and disease progression, even in the presence of HVT transmission.

Follow-up studies would be useful to show clearly whether vaccine transmission is a missing link in the chain of cause and effect in this and other systems, and we now highlight this in the manuscript (lines 114-121 and lines 438-455). Nevertheless, even without this additional research, the downstream impacts and their causes that we highlight remain (1) previously unreported, (2) important for understanding virus management and dynamics, and impacts on hosts, and (3) unaccounted for in many epidemiological models, including all existing models for Marek’s disease.

“To exclude this likely scenario that undermines their conclusions, the authors should perform the following experiments.”

**Response**: For the reasons stated above, putative HVT transmission does not undermine our conclusions regarding the strong downstream effects of vaccination, or the effect of reduced pathogen viral load on disease progression.

Given the expected absence of HVT transmission based on the results of previous experiments (see our response to reviewer 2), we respectfully disagree that HVT transmission was likely *a priori* in our experiments.

Regarding the suggested additional experiments below, while they may be useful as a follow-up, we do not believe their absence undermines our study. We further believe that the time and cost required is not warranted as part of this existing study, especially given the lack of evidence in our new results that vaccine transmission is a primary causal factor. We address this further under each point below.

“a.	Since the contact animals could have acquired HVT, the authors should test the blood (and feathers) of the contact animals for HVT genome copies by qPCR.”

**Response**: We thank the reviewer for this suggestion and have analysed HVT genome copies in a subsample of 89 blood samples. Please see our responses above on this same issue. 91% of contacts of vaccinated birds in our subsample were negative for HVT in buffy coat from our additional qPCR study. These results do not support HVT transmission as the defining reason behind the downstream effects. If we had found more extensive evidence of HVT transmission in our subsample, we would have quantified HVT in all contact individuals and incorporated this into our process analysis.

“b.	In addition, they should test if the dust contains HVT genome copies, as even HVT antigens released into the environment could have some protective effect in the contact animals and explain the outcome of their animals experiment.”

**Response**: We did not collect dust samples in this experiment, but our new results suggest that this is now redundant. We believe it is safe to assume that low levels of HVT were deposited in the environment, given that some transmission occurred.

“c.	The authors could also use a spread deficient HVT vaccine to proof that spread of HVT is not responsible for the observed phenotype.”

**Response**: This is a smart idea, but we are not aware of the existence of a spread-deficient HVT strain. As mentioned above, HVT is typically assumed to be naturally spread-deficient.

Given the limited but non-zero transmission of HVT in our experiments, we acknowledge that such a study would be worthwhile to disentangle the effects of shedder vaccination on the transmitted pathogen dose from those of vaccine transmission, which is particularly relevant for other MD vaccines (e.g. Rispens) that are known to be highly transmissible. However, this was beyond the scope of our study.

“2)	Along those lines. It would be worth testing if contact animals of HVT vaccinated animals (mock infected) also show reduced clinical symptoms when they are challenged with MDV.”

**Response**: We acknowledge that this would further help to quantify the effect of vaccine transmission on contact bird response to infection, and will plan to incorporate this in future studies. However, this was not the focus of our current study. Neither this nor the experiment described in c. above are essential for the validity or general importance of our study.

“3)	The authors claim that the reduced shedding of MDV from HVT vaccinated animals contributes to the reduced clinical symptoms in the contact animals. However, very few plaque forming units are sufficient to induced disease and was shown by various laboratories. The authors previously demonstrated that cohousing infected and uninfected animals for 4 hours is sufficient for transmission, indicating that not massive amounts of virus are needed to infect contact animals. They cohoused animals for 48 hours, which would be more than enough despite the lower shedding levels. The authors should consider and discuss these aspects in their manuscript.”

**Response**: Our new nested analysis results reinforce our conclusion that increasing pathogen load leads to more severe symptoms. Few plaque-forming units may be sufficient to sometimes induce disease, but our results clearly show that symptoms are more prevalent and more severe, and mortality is higher, with higher pathogen loads. Furthermore, previous studies of birds inoculated with differing doses of MDV have revealed more severe symptoms with higher inoculation dose (see lines 86-88 and 470-472 where we refer to these earlier studies). Hence a dose response is to be expected.

The reviewer makes a good point regarding the 4-hour contact duration pilot study, in which most contact birds developed MD symptoms within 8 weeks (only 2/16 showed no clinical signs). Unfortunately, we did not collect viral load data from these early pilot experiments. On the other hand, it is not clear whether absolute quantities shed from vaccinated individuals in 48 hours – with their mostly extremely low feather viral loads – would be more or less than quantities shed by highly virus-laden unvaccinated birds in 4 hours. Given the lack of FVL data for the 4-hour experiment, rendering all conclusions as guesswork, we have not added discussion of this specific comparison to the revised manuscript.

“Minor Points:

1) Virus remains infectious – not ‘viable’ “

**Response**: Line 85 “viable” changed to “infectious”

2) “Line 128: Shedder experiments are common in the field. So stating that they used a novel setup is overstating it.  “

**Response**: We have deleted the opening of this sentence, “Given the novelty of the experimental setup, …”, leaving the sentence reading “Appropriate contact duration and sampling times to examine shedder vaccination effects needed to be established in pilot experiments prior to the main trial.” (lines 207-208).

3) “Why did the authors not use a more current vaccine like SB-1 or CVI988? And why did they use a low virulent virus instead of MD5 or vv+ strains for their experiments? The authors should discuss these points. “

**Response**. We now address this in the final paragraph of the Introduction, lines 172-177. The text reads:

“We chose HVT vaccine due to its low transmissibility, its wide usage both to combat MDV and as a vector vaccine, and due to our extensive previous experience with this vaccine allowing optimization of experimental methods. We chose to focus solely on a well-studied vMDV (rather than more virulent vvMDV or vv+MDV) pathogen strain to allow comparison with many past studies, and to maximize replication and hence our ability to detect differences in downstream effects”.

We already highlight in the Discussion the need for additional studies extending to other vaccines and more virulent virus strains (lines 559-565).

**References**

Cho BR. Horizontal transmission of turkey herpesvirus to chickens. 5. Airborne transmission between chickens. Poultry science. 1976 Sep;55(5):1830-3.

Cho BR, Kenzy SG. Horizontal Transmission of Turkey Herpesvirus to Chickens: 2. Some Factors Affecting Transmission. Poultry science. 1973 Mar 1;52(2):608-13.

Cho BR, Kenzy SG. Horizontal Transmission of Turkey Herpesvirus to Chickens: 3. Transmission in Three Different Lines of Chickens. Poultry Science. 1975 Jan 1;54(1):109-15.

Islam, A. F. M. F.; Groves, P. J.; Underwood, G. J.; Walkden-Brown, S. W., Dynamics of Marek's disease virus and herpesvirus of turkey shedding in feather dander of broiler chickens. In Poultry Research Foundation: Sydney, 2005; pp 105-108.

Islam, A.; Walkden-Brown, S. W., Quantitative profiling of the shedding rate of the three Marek's disease virus (MDV) serotypes reveals that challenge with virulent MDV markedly increases shedding of vaccinal viruses. J Gen Virol 2007, 88, (Pt 8), 2121-8.
