# Supplementary material for: Pathogen transmission from vaccinated hosts can cause dose-dependent reduction in virulence
Source: PLoS Biol. 2020 Mar 5;18(3):e3000619. doi: 10.1371/journal.pbio.3000619 (PMC7058279; doi:10.1371/journal.pbio.3000619)
Supplement: Attachment — Submitted filename: PBIOLOGY-D-19-02539R2_ResponseToReviewers_07Jan2020.docx [file pbio.3000619.s011.docx]

Response to reviewers for manuscript PBIOLOGY-D-19-02539R2 entitled “**Pathogen transmission from vaccinated hosts can cause dose-dependent reduction in virulence**”.

Dear Dr Richardson,

Please find below our response to the reviewer comments. Responses begin with the word “Response” in bold. Text from the original PLoS Biology decision email is within quotation marks.

We thank the editors and reviewer for their highly constructive contributions to improving this manuscript.

---------------

“Reviewer #3:

General Comment:

In this manuscript, the authors investigated the effect of leaky vaccines on the transmission of an avian herpesvirus to unvaccinated contact chickens. Their data suggests that the reduced replication of Marek's disease virus (MDV) in vaccinated animals has a protective effect on the contact chickens. In this resubmission of the manuscript PBIOLOGY-D-19-02539, the authors addressed most of the reviewer's comments and improved the manuscript, which is now suitable for publication.

Minor Points:

1)	Line 364: it should be "91% of contact birds" and not 90%.”

**Response**: We have changed “90%” to “91%” (line 364).

We have also made a few small changes to the Discussion text to improve clarity, with no changes to meaning. In particular, we have re-worked the paragraph on the potential impacts of our findings on pathogen virulence evolution (LINES 425-445).

We have carried out all changes requested in the PLOS Formatting Requests document.

On behalf of all the authors, sincerely,

Richard Bailey.
